# Four Decades of Cytochrome P450 Research in Africa: A Historical Overview of Scientific Contributions, Research Trends, and Future Directions

**DOI:** 10.3390/biology15151242

**Published:** 2026-07-28

**Authors:** Khajamohiddin Syed

**Affiliations:** 1 Department of Biochemistry and Microbiology, Faculty of Science, Agriculture and Engineering, University of Zululand, Empangeni 3886, South Africa; syedk@unizulu.ac.za; Tel.: +27-035-902-6857; 2 National Institute for Theoretical and Computational Studies (NITheCS), Stellenbosch 7600, South Africa

**Keywords:** Africa, cytochrome P450 monooxygenases, insecticide resistance, microbial biotechnology, steroidogenesis, pharmacogenomics

## Abstract

Cytochrome P450 monooxygenases (CYPs/P450s) are versatile heme-containing enzymes involved in drug metabolism, steroidogenesis, secondary metabolite biosynthesis, and biotechnology. Although African researchers have contributed to P450 research for nearly four decades, no comprehensive assessment of African-led research has been conducted. This study presents a historical and bibliometric analysis of African-led P450 research published between 1987 and 2025 using PubMed and Web of Science. Of 1382 retrieved articles, 558 had African corresponding authors, and 149 included African co-authors. Research output increased markedly after 2011, with South Africa contributing 64% of African-led publications. Corresponding authors represented 31 African countries, while collaborations extended to 54 non-African countries, demonstrating strong international integration. Drug metabolism, pharmacology, and toxicology dominated the research landscape, followed by molecular biology, genomics, enzymology, and structural biology. African researchers have made important contributions to pharmacogenomics, steroidogenesis, insecticide resistance, microbial biotechnology, biosensors, and natural-product metabolism. Collectively, African-led publications have received more than 20,500 citations on Google Scholar, highlighting their global scientific impact. Despite this progress, important gaps remain in large-scale pharmacogenomics, structural biology, systems biology, industrial biotechnology, and omics-based studies. Overall, African researchers have made substantial contributions to global cytochrome P450 research and have created opportunities for future scientific growth and collaboration.

## Simple Summary

Cytochrome P450 enzymes are important proteins found in almost all living organisms. They help break down medicines, produce hormones, detoxify harmful substances, and produce many useful natural compounds. Although African scientists have been studying these enzymes for nearly 40 years, no study has previously examined the overall contribution of African researchers to this field. This study analyzed scientific publications produced by African researchers between 1987 and 2025. The analysis showed that African scientists published more than 550 research articles as lead authors, with research activity increasing rapidly after 2011. South Africa contributed the largest share of publications, while researchers from 31 African countries participated in this research area. African scientists made important contributions to understanding drug responses, hormone production, insecticide resistance in disease-carrying mosquitoes, microbial biotechnology, and enzyme-based biosensors. The study also revealed strong international collaborations between African and non-African researchers. However, important gaps remain in precision medicine, advanced biological data analysis, and industrial biotechnology. By identifying strengths, research gaps, and opportunities for collaboration, this study can help guide future scientific investment and innovation that may improve healthcare, biotechnology, agriculture, and environmental sustainability across Africa.

## 1. Introduction

Cytochrome P450 monooxygenases (CYPs/P450s) are heme-containing mixed-function oxidoreductases ubiquitously distributed across the domains of life, including viruses [1]. P450s were discovered more than six decades ago and were named “cytochrome P450 monooxygenases,” as the term “cytochrome” denotes a heme-containing protein, while “P450” refers to the characteristic absorption peak observed at 450 nm when the reduced enzyme is bound to carbon monoxide. Finally, “monooxygenase” signifies the enzyme’s ability to incorporate a single atom of molecular oxygen into a substrate [2,3,4,5]. A classification and nomenclature system was developed to categorize P450s. Members of the same family share >40% amino acid sequence identity, while members of the same subfamily share >55% amino acid sequence identity. In the naming format, each P450 is abbreviated as CYP, followed by an Arabic numeral for the family, a letter for the subfamily, and a final Arabic numeral for the individual gene (e.g., CYP1A1) [1,6,7]. This classification system is now well-established, and a large number of P450s from different species across domains and viruses, with their family and subfamily assignments, are available in the P450ATLAS database [1].

Due to their exceptional stereoselectivity and regioselectivity, diverse catalytic mechanisms, and substrate promiscuity, P450 enzymes have become indispensable across several fields. They are widely utilized in the production of fine chemicals [8] and fragrances [9,10], drug discovery and development [11,12], pharmaceutical compound production [13], generation of biofuels [14], biosensing applications [15], and bioremediation [16,17]. One of the primary and well-established roles of P450s is in pharmacogenomics, where P450s’ genetic polymorphisms were found to play a key role in drug metabolism [11,12]. P450s were also found to play a key role in the synthesis of valuable secondary metabolites in various organisms, where they perform stereo- and regio-specific oxidation, thereby contributing to metabolite diversity [18,19].

Africa represents a particularly important setting for P450 research because the continent harbors the greatest human genetic diversity worldwide, carries a substantial burden of infectious and non-communicable diseases, and possesses exceptional biodiversity that provides valuable natural products for drug discovery and biotechnology. Understanding African P450 research is therefore essential for advancing pharmacogenomics, precision medicine, biocatalysis, agricultural innovation, and environmental sustainability while ensuring that African populations and biological resources are appropriately represented in global scientific research.

Researchers from Africa, with 54 countries according to the United Nations, also focused on these enzymes. The first article from Africa on P450 enzymes was published in 1987 by Prof. Joshua Joachim Taljaard’s research group at the University of Stellenbosch, South Africa, who identified differences in substrate specificity in microsomes prepared from rat brain and liver and suggested that this was due to different profiles of P450 isozymes in these two organs [20]. To date, many studies on P450 enzymes have been published by African researchers covering diverse topics, including drug metabolism, pharmacogenomics, steroidogenesis, insecticide resistance, microbial biotechnology, structural biology, and biocatalysis. Despite these important contributions, no comprehensive bibliometric and thematic assessment has systematically evaluated the development, scientific impact, collaboration patterns, research priorities, and future directions of African-led P450 research. Therefore, this study presents a comprehensive analysis of African-led P450 publications indexed in PubMed and Web of Science between 1987 and 2025. Specifically, the study aims to: (i) characterize temporal publication trends and the growth of African P450 research; (ii) examine the geographic distribution of research output and national and international collaboration patterns; (iii) identify the major thematic research areas and highlight the scientific contributions of African researchers; and (iv) identify existing research gaps and future opportunities to guide the continued development of P450 research across Africa.

## 2. Materials and Methods

### 2.1. Data Source and Search Strategy

A literature search was conducted in PubMed and Web of Science using a search strategy that combined controlled vocabulary and free-text terms related to P450 enzymes and Africa. The following search terms were used: “cytochrome P450 and P450 and Africa”. The search covered articles published until 2025. The search strategy was designed to identify African-led P450 research associated with African researchers, institutions, or populations. Although every effort was made to comprehensively capture the literature through PubMed and Web of Science searches and manual screening, some relevant publications may have been inadvertently missed due to database indexing practices or differences in terminology. This limitation is inherent to bibliometric studies and does not affect the overall trends and conclusions presented herein.

### 2.2. Eligibility Criteria, Study Selection, and Classification Process

All articles retrieved from PubMed and Web of Science were included in the analysis. Articles overlapping in the Web of Science and PubMed databases were excluded. Titles, abstracts, and full texts of the articles were manually reviewed for the presence of P450 aspects, and researchers were classified by their institutional affiliations, as reported in the published articles, which also included their nationalities. Corresponding authorship was used solely as an operational, reproducible criterion for identifying African-led studies and should not be interpreted as an absolute measure of scientific leadership. All hit articles were then classified into the following five categories:

1. Articles where African researchers are corresponding authors (Table S1).

2. Articles where African researchers are co-authors and non-African P450 researchers are corresponding authors (Table S2).

3. Articles where non-African researchers are associated with African universities (Table S3).

4. Articles that have no P450 research aspects (Table S4).

5. False positive—Non-African researchers working on African problems where P450 aspects are involved (Table S5).

**Figure 1:**
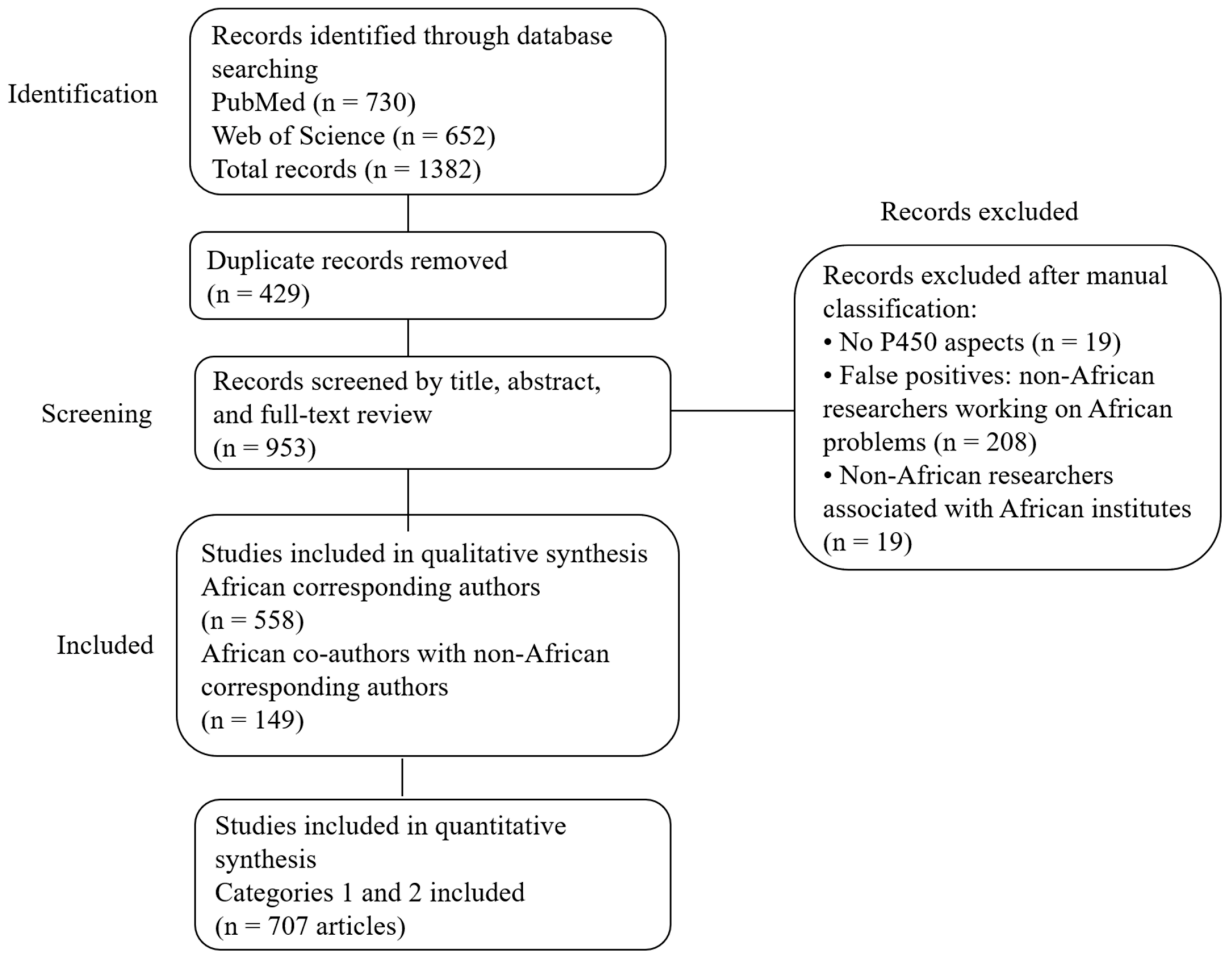
A PRISMA flow diagram summarizing the literature search, screening, eligibility assessment, and inclusion process for African-led P450 research articles. Records were retrieved from the PubMed and Web of Science databases and manually screened using predefined inclusion and exclusion criteria. Articles were classified based on authorship, relevance to P450 research, and the involvement of African researchers. Only studies involving African corresponding authors or African co-authors were included in the quantitative synthesis.

Only categories 1 and 2 were included in the quantitative synthesis presented in the study. The literature search, screening, eligibility assessment, and study inclusion process were summarized using a PRISMA-style flow diagram (Figure 1) following PRISMA 2020 reporting guidelines [21].

### 2.3. Data Extraction

For each included article, the full-text PDF was examined to extract:

- Corresponding author and institutional affiliation;
- Country of origin;
- Co-authorship and international collaboration patterns;
- Journal of publication and year;
- Primary research focus.

This manual verification was necessary because metadata displayed in PubMed or Web of Science abstracts may be incomplete or inconsistent. These procedures were intended to maximize retrieval of African-led P450 publications but do not imply absolute completeness of the dataset. The findings should therefore be interpreted as a comprehensive historical overview of the currently retrievable literature.

### 2.4. Data Analysis and Visualization

The extracted data were compiled and analyzed using Microsoft Excel. Descriptive statistics were used to summarize publication trends, country-level contributions, author productivity, and collaboration networks. The African continent map is generated using MapChart (https://www.mapchart.net/). Collaborations between African and non-African researchers were presented as heat maps, with red squares indicating collaboration and green squares indicating no collaboration. To generate the heat maps, collaboration between countries was included in a tab-delimited file, which was then exported to the multi-experiment viewer (MeV) program [22]. In the program, +3 was chosen to indicate the presence of a collaboration (red), and −3 to indicate its absence (green). Then, the analysis was performed using hierarchical clustering with the Euclidean distance metric. The resulting heat map was presented, with countries on both axes.

### 2.5. Quality Assurance

The search strategy using the predetermined keywords retrieved all publications associated with the author from both PubMed and Web of Science, suggesting that publications by African researchers were comprehensively captured, as the search results also considered affiliation, geographic location, and keywords in the articles. Notably, the retrieved dataset included: Table S1: 558 articles in which African researchers were corresponding authors; Table S2: 149 articles in which African researchers were co-authors, indicating that publications containing African institutional affiliations were also captured; Table S3: 19 articles authored by non-African researchers affiliated with African institutions and conducting P450 research; Table S5: 208 articles authored by non-African researchers addressing African health and biological problems involving cytochrome P450s. The presence of these diverse categories of publications strongly suggests that the search results were not restricted to articles explicitly mentioning “Africa” in their titles, abstracts, or keywords, but also captured publications associated with African institutions and affiliations. Every effort was made to identify, screen, and classify all relevant publications using predefined search criteria and manual full-text verification. Nevertheless, because the study relied on manual curation rather than an independently validated reference dataset, some relevant publications may have been unintentionally omitted or differently classified owing to database indexing practices, incomplete affiliation information, or differences in terminology. Consequently, the dataset should be interpreted as a comprehensive historical overview of the currently retrievable African-led P450 literature rather than an exhaustive census of all African P450 publications.

### 2.6. Research Clustering, Thematic Analysis, and the Use of Generative AI

African-led P450 publications were grouped into thematic research clusters based on study objectives, biological systems, and application areas following the full-text review. Because individual articles often addressed multiple research themes, studies were allowed to contribute to more than one cluster. Consequently, cumulative percentages across clusters exceed 100%. The author used ChatGPT (OpenAI, GPT-5.3) and Grammarly Premium during manuscript preparation for thematic classification, language editing, formatting assistance, and graphical abstract generation. For thematic classification, articles were uploaded to ChatGPT for initial classification based on the work, and then all articles were manually checked for allocation to different thematic clusters. The author takes full responsibility for the manuscript’s content, and all AI-generated outputs were critically reviewed and validated.

### 2.7. Citation Impact Analysis

To complement the descriptive bibliometric assessment, the scientific impact of African-led P450 research was evaluated using Google Scholar citation data. Google Scholar was selected because it provides broad coverage of peer-reviewed publications across multiple publishers and scientific disciplines.

For each publication included in the study, the total number of Google Scholar citations was recorded (Table S6). Since citation accumulation is strongly influenced by publication age, two additional citation-based indicators were calculated to enable fairer comparisons across publications from different years.

First, citation velocity (citations per year) was calculated as: Citation Velocity = Total Google Scholar Citations/Years Since Publication, where Years Since Publication = 2026 − Publication Year + 1.

Citation velocity provides an estimate of the average number of citations accumulated per year since publication. It therefore reduces bias associated with older publications having had more time to accumulate citations.

Second, a year-normalized citation impact (YNCI) was calculated to account for annual differences in citation opportunities. For each publication year, the mean citation count of all African-led P450 publications published during that year was calculated. The YNCI for each article was then determined as:

YNCI = Total Google Scholar Citations/Mean Citations per Publication Year.

where the mean citations per publication year were calculated as:

Mean Citations per Publication Year = Total Citations by Publication Year/Number of Publications in the Year.

A YNCI value greater than 1 indicates that a publication has received more citations than the average African-led P450 publication published during the same year. In contrast, values below 1 indicate below-average citation performance. Descriptive statistics, including total citations, citation velocity, and YNCI distributions, were calculated using Microsoft Excel. Detailed citation metrics for all 558 African-led cytochrome P450 publications, including Google Scholar citations, citation velocity, and year-normalized citation impact (YNCI), are provided in Table S6.

### 2.8. Study Limitations

This study was designed as a historical and descriptive bibliometric overview of African-led cytochrome P450 research rather than a comprehensive scientometric investigation. Consequently, advanced citation-network analyses, co-authorship network analyses, and keyword evolution studies were beyond the scope of the present work and represent important directions for future research.

In addition, although all publications were screened and classified using predefined criteria and manual full-text verification, thematic classification was performed by a single investigator and was not independently validated by a second reviewer. Therefore, some degree of subjective interpretation is unavoidable, and a small number of publications could reasonably be assigned to alternative thematic categories. Likewise, despite comprehensive searches of PubMed and Web of Science, some relevant publications may have been unintentionally omitted because of database indexing practices, affiliation inconsistencies, or differences in terminology. These limitations should be considered when interpreting the thematic distribution presented in this study.

## 3. Results and Discussion

### 3.1. P450s Research Is Gaining Momentum in Africa

A PubMed and Web of Science search with the terms “cytochrome P450 and P450 and Africa” yielded a total of 1382 articles: 730 from PubMed and 652 from Web of Science from 1987 to 2025. Among these articles, 429 are duplicates as they are retrieved from both databases. For 558 articles African researchers are corresponding authors, and the research involved P450 enzymes (Figure 1 and Table S1); for 149 articles African researchers co-authored while non-African researchers were corresponding authors (Table S2); there were 19 articles where non-African researchers were associated with the African country institute (Table S3); African researchers published 19 articles which had no P450 aspects (Table S4); and 208 articles are false positives where non-African researchers were working on African problems where P450 aspects are involved (Table S5). Detailed information on articles across different categories from PubMed and Web of Science is presented in Figure 1.

**Figure 2:**
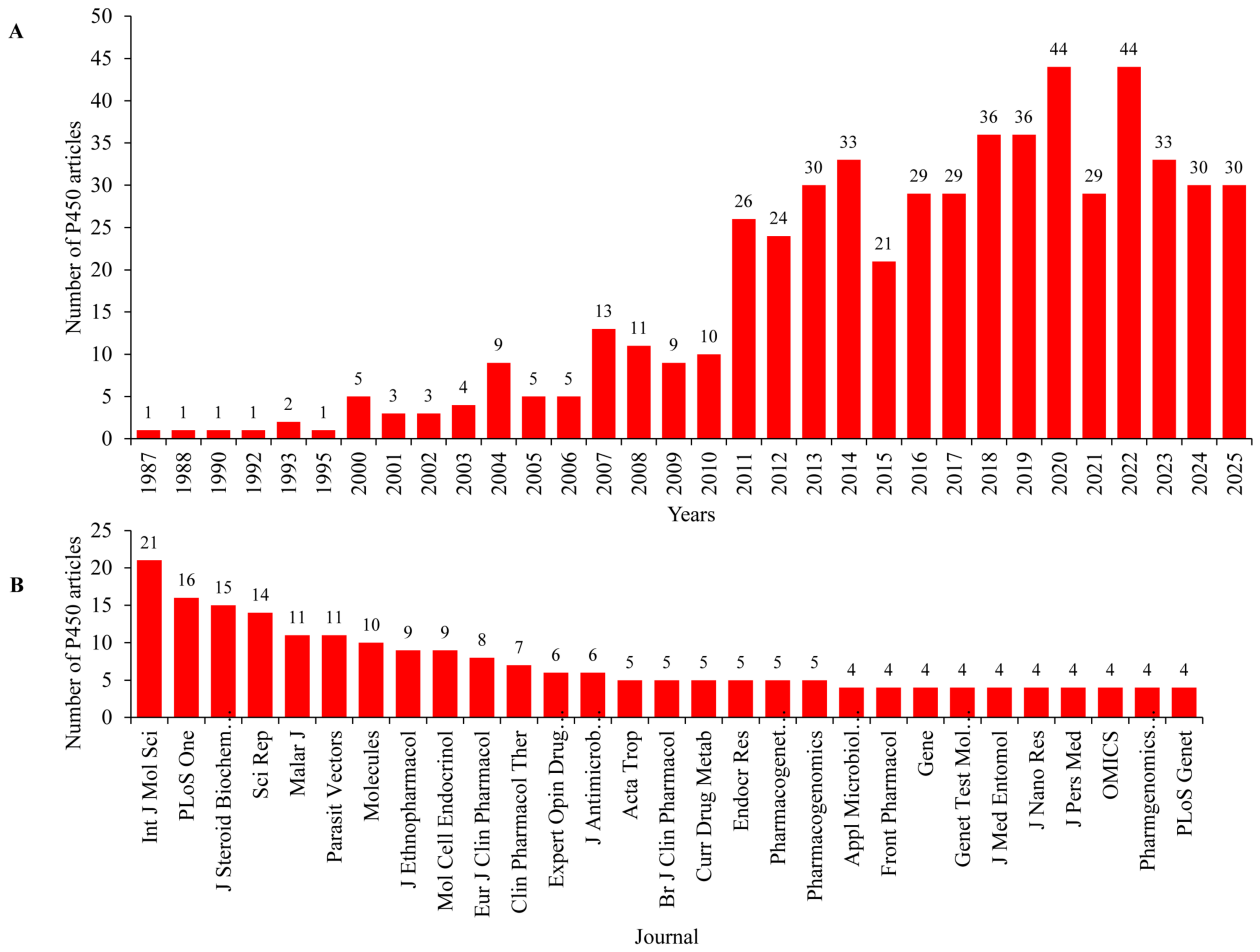
Publication trends and journal distribution of African-led P450 research. (**A**) Year-wise distribution of African-led P450 research articles published between 1987 and 2025. (**B**) Journal-wise distribution of African-led P450 research articles. Only journals publishing five or more P450-related articles are included in the figure. The numbers above the bars indicate the number of articles published in a given year (**A**) or in a given journal (**B**). Detailed publication data are provided in Table S1.

As indicated in the introduction, the first article on P450 research was published in 1987 by Prof Taljaard (corresponding author) from the University of Stellenbosch, South Africa [20] (Table S1). From 1987 to 2010, 84 articles, representing ~12% of the total, were published; from 2011 onwards, the number of P450 articles increased sharply, and in 2020 and 2022, the highest number, 44 articles, was published (Figure 2A and Table S1). This indicates that P450 research in Africa gained momentum in 2011. The 558 articles were published in 298 journals (Table S1), indicating a diverse range of outlets in which African researchers publish their findings. However, among 298 journals, a handful of 19 journals to be mentioned have five or more articles: the International Journal of Molecular Sciences has the highest number of P450 articles (21), followed by PLoS ONE (16 articles), Journal of Steroid Biochemistry and Molecular Biology (15 articles), Scientific Reports (14 articles), Parasites & Vectors and Malaria Journal (11 articles) and Molecules (10 articles) (Figure 2B and Table S1). The increase in publication output was accompanied by substantial scientific impact, as demonstrated by the citation analyses presented in Section 3.6.

### 3.2. African P450 Researchers Are Well Integrated Globally

**Figure 3:**
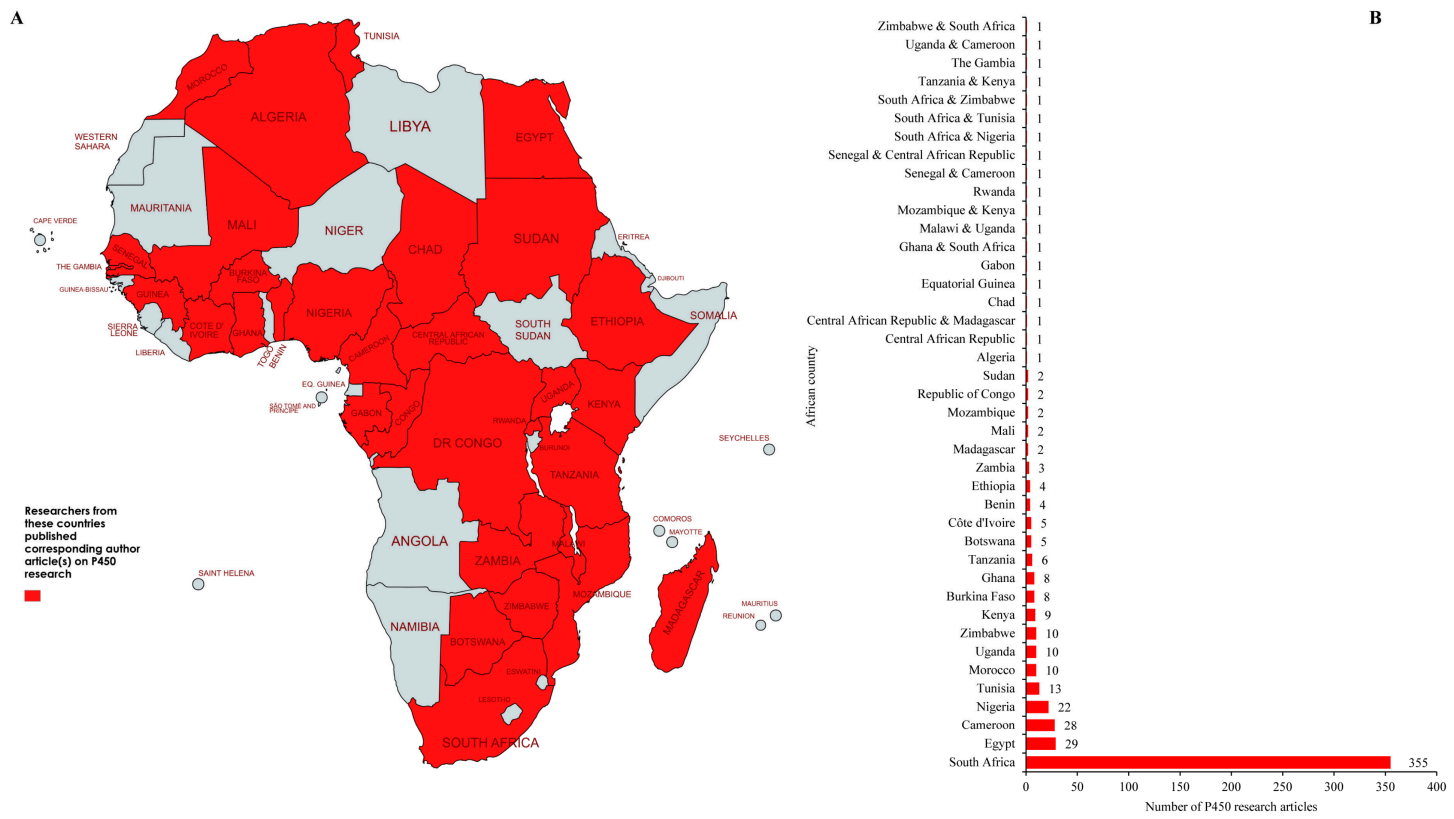
The geographic distribution of African-led P450 research across the African continent. (**A**) A map of Africa showing countries from which African-led P450 research articles have been published; countries that have contributed P450 publications are highlighted in red. (**B**) The number of African-led P450 research articles published by each African country. The numbers next to the bars indicate the total number of P450-related articles contributed by the respective country. Detailed country-wise publication data are provided in Table S1.

Among 54 African countries, researchers from 31 have published P450 articles as corresponding authors (Figure 3A), indicating that researchers across the African continent are involved in P450 research. Among 31 African countries, researchers from South Africa published the most articles (355), followed by Egypt (29), Cameroon (28), Nigeria (22), and Tunisia (13) (Figure 3B). This indicates that South African researchers contributed 64% of P450 articles from the African continent.

**Figure 4:**
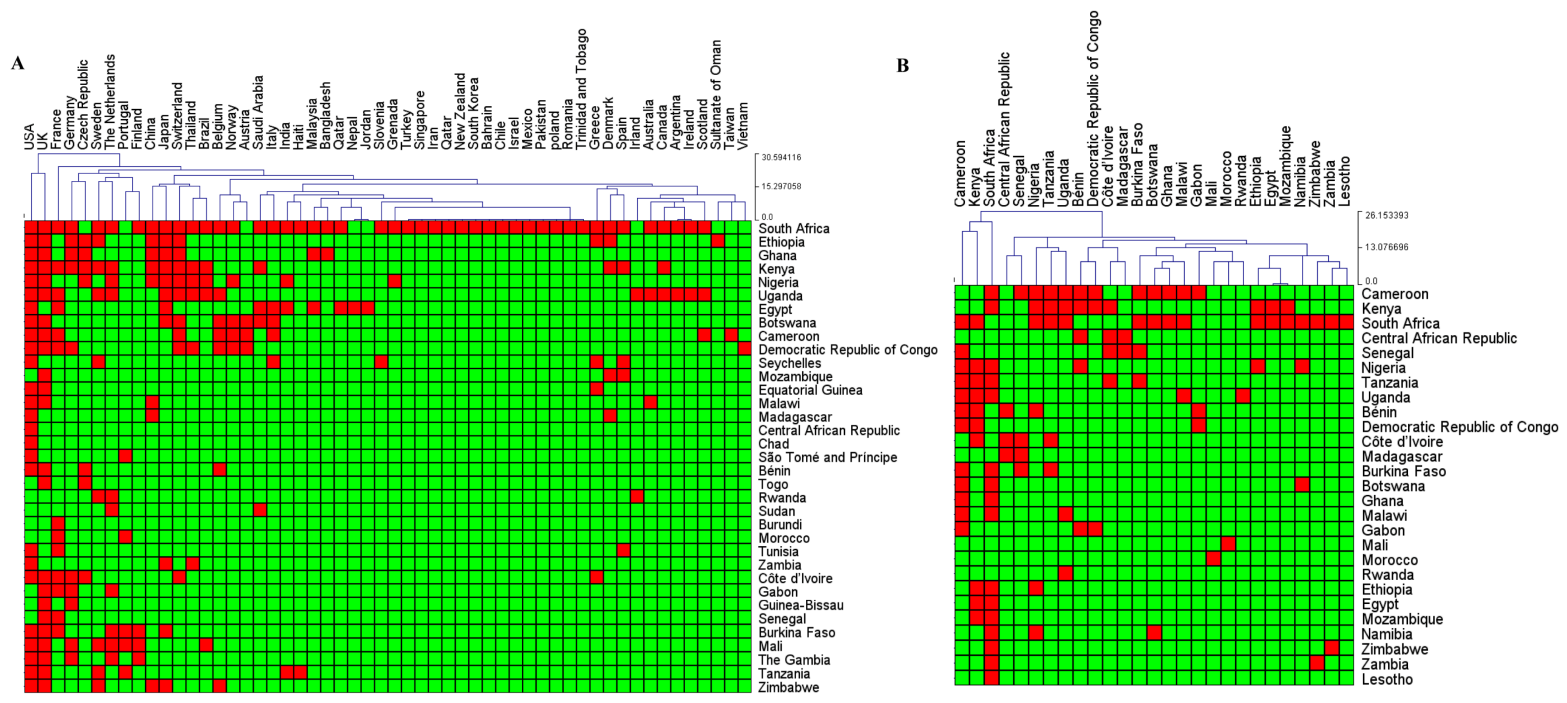
A heat map representation of collaboration networks among African P450 researchers and international collaborators. A heat map showing collaboration patterns of African P450 researchers with international researchers (**A**) and among African researchers (**B**). The red squares indicate research collaborations/co-authored publications between countries, whereas the green squares indicate no detected collaborations. Hierarchical clustering was performed using the Euclidean distance metric to visualize collaboration patterns and research networking among countries involved in African P450 research. Countries are represented on both axes of each heat map.

African P450 researchers are well connected to researchers worldwide, as evidenced by collaborations with researchers from 54 non-African countries (Figure 4A and Table S2). Among non-African countries, researchers from the UK and the USA have the most collaborations with researchers in 26 and 23 African countries, respectively (Figure 4A). South African P450 researchers established collaborations with researchers from 45 non-African countries, the highest among African countries, followed by Kenya and Uganda (16 each), Nigeria (12), Ethiopia (11), and Egypt (10) (Figure 4A).

P450 researchers from 27 African countries established collaborations with colleagues from other African countries (Figure 4B). Among African countries, P450 researchers from South Africa collaborated with the most African P450 researchers (16), followed by Cameroon (12) and Kenya (10) (Figure 4B). This further highlights the worldwide outreach of South African P450 researchers. A small number of non-African researchers are affiliated with African universities while maintaining appointments at their home institutions (Table S3). Researchers Yoshinori Ikenaka and Shouta M. M. Nakayama from Hokkaido University, Japan, have long-term affiliations with North-West University, South Africa, and the University of Zambia, Zambia (Table S3). This distribution is consistent with the broader scientific publication landscape in Africa, where South Africa has consistently been the leading contributor to research output owing to its well-established research infrastructure, funding, and higher education system.

Although much of the information provided in Tables S2 and S3 on collaboration is included, the in-depth analysis in future work will include additional network analyses, such as network centrality, collaboration strength, and community detection.

### 3.3. South African Researchers Are Major Contributors Among African P450 Researchers

**Figure 5:**
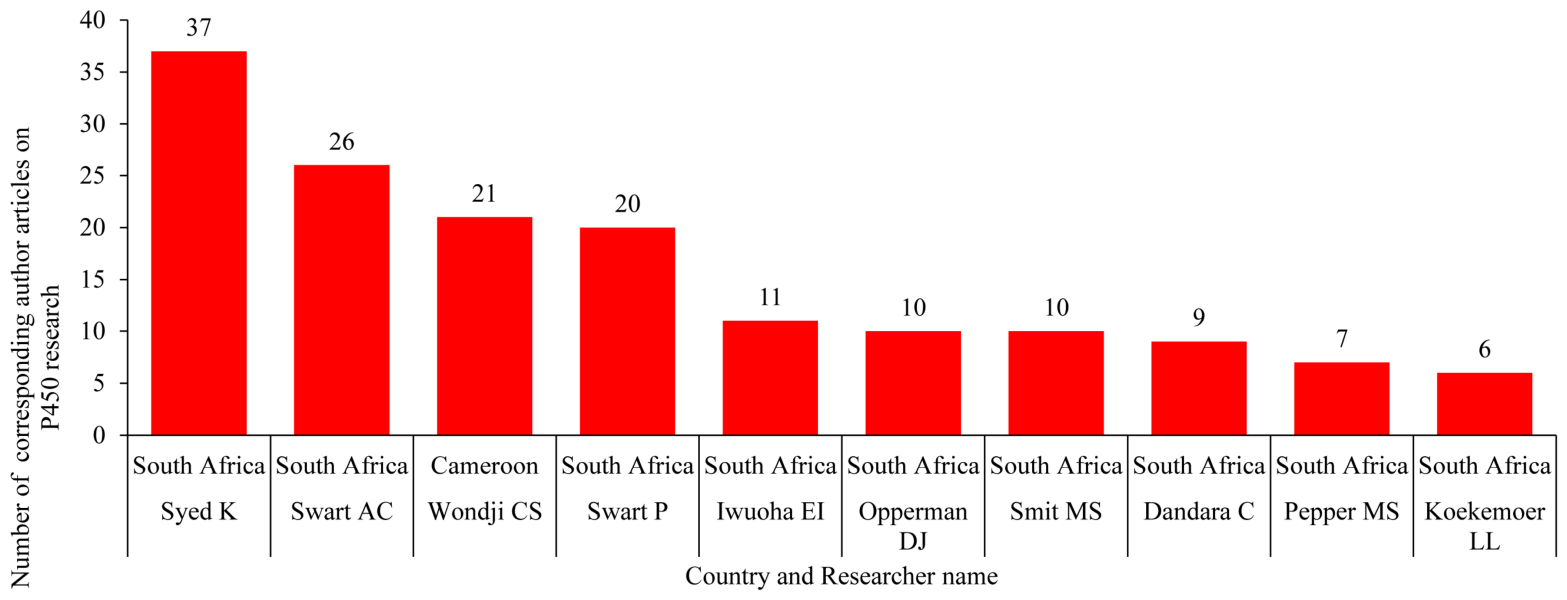
The contributions of leading African researchers to cytochrome P450 research. The figure presents the number of African-led P450 research articles published by leading African researchers as corresponding authors. The numbers above the bars indicate the total number of corresponding-author P450 publications attributed to each researcher. Researchers with affiliations in more than one African country are listed under their current country of affiliation. Publication counts include all African-led P450 articles published throughout their careers, regardless of prior African institutional affiliations. Detailed information on researcher contributions is provided in Table S1.

Analysis of P450 researchers revealed that 328 are based in Africa (Figure 5 and Table S1). Among these P450 researchers, the highest number is from South Africa (166), followed by Egypt (26), Nigeria (23), Tunisia (14), Cameroon and Kenya (12), and Morocco (11) (Table S1), indicating that nearly half of African P450 researchers are from South Africa. Analysis of African P450 researchers revealed that only a handful are established and consistently publish research articles on P450 enzymes (Figure 5). In total, 10 P450 researchers have published >5 corresponding-author articles on P450 aspects (Figure 5). This indicates that P450 research represents one of several research interests for most African researchers rather than their primary area of investigation. Among these 10 researchers, nine are from South Africa, and one is from Cameroon (Figure 5). This indicates that among African researchers, South African researchers are firmly established in P450 research. To illustrate the breadth of African contributions across the major thematic areas identified in this bibliometric analysis, representative examples of scientific contributions from selected researchers are summarized in Table 1. The researchers were selected to illustrate sustained contributions to different areas of African P450 research; the table is illustrative and is not intended to represent a ranking or an exhaustive list of contributors.

### 3.4. Non-African Researchers Showed Enormous Interest in African P450 Research

A large number of studies (208) were found to be false positives, with non-African researchers as authors, revealing that a significant portion of these studies focus on African populations from Ghana, Zimbabwe, Sudan, West Africa, and sub-Saharan Africa (Table S5). These studies focus either on pharmacogenetics or on disease vectors prevalent in Africa, such as *A. gambiae*, *A. funestus*, and *A. arabiensis*, or on assessing herb–drug interactions of African medicinal plant extracts (Table S5). This suggests that non-African researchers are also working to address health and biological challenges affecting African populations.

### 3.5. Thematic Distribution and Research Trends in African P450 Studies

A thematic analysis of the 558 retrieved P450-related publications identified multiple major research categories across African P450 research (Table 2). Drug metabolism, pharmacology, and toxicology represented the dominant area, accounting for approximately 40% of all publications. These studies primarily focused on xenobiotic metabolism, hepatotoxicity, pharmacokinetics, and P450-mediated drug interactions. Molecular biology and genomics-related studies formed the second-largest category (~25%), with a strong emphasis on P450 gene expression, polymorphism analysis, evolutionary studies, and recombinant expression systems. Biochemical and enzymological investigations accounted for approximately 16–18% of publications and largely addressed catalytic mechanisms, ligand binding, heme chemistry, and structure–function relationships. Smaller but important research areas included vector biology and insecticide resistance (~5–6%), microbial and fungal P450 biotechnology (~4–5%), environmental toxicology (~2–3%), and plant/natural-product metabolism (~2–3%). The analysis further revealed that relatively few studies focused on clinical pharmacogenomics, systems biology, structural biology, industrial biotechnology, or large-scale omics approaches.

Overall, African P450 research is strongly centered on biomedical and toxicological applications, particularly drug metabolism and xenobiotic detoxification. This trend likely reflects the significant health burden of infectious and chronic diseases across the continent. Future analyses of keyword evolution and thematic shifts would provide valuable complementary information.

### 3.6. Citation Impact Demonstrates the Scientific Influence of African-Led P450 Research

Although publication output indicates research productivity, citation analysis provides additional insight into the scientific influence and visibility of published work. Citation analysis of the 558 African-led P450 publications included in this study revealed that these articles collectively accumulated 20,528 Google Scholar citations, corresponding to an average of 36.8 citations per publication and a median of 21 citations (Table S6). The most highly cited publication received 465 citations, demonstrating that several African-led studies have achieved substantial international recognition and have become influential references within the global P450 research community.

Because citation counts naturally increase with publication age, citation velocity was calculated to normalize citation accumulation over time. The average citation velocity across all publications was 3.56 citations per year, while the median citation velocity was 2.27 citations per year. The highest-performing publication accumulated 34 citations per year, indicating sustained scientific interest and continued relevance after publication. These findings demonstrate that African-led P450 research has not only increased in publication output but has also generated studies that continue to attract consistent scientific attention.

To further account for differences in publication year, the year-normalized citation impact (YNCI) was calculated by comparing each publication’s citation count with the mean citation count of publications from the same year. Among the 558 publications, 177 articles (31.7%) achieved a YNCI greater than 1, indicating citation performance above the annual average. Furthermore, 65 publications (11.6%) received at least twice the average citation impact of publications from the same year, while 29 publications (5.2%) achieved more than three times the annual average citation impact. These observations indicate that a considerable proportion of African-led P450 publications have performed substantially better than expected relative to their publication year, suggesting that many studies have had broad scientific influence beyond simply contributing to publication numbers.

The citation analysis also demonstrates that African P450 research has produced numerous highly influential publications spanning pharmacogenomics, steroidogenesis, insecticide resistance, microbial biotechnology, structural biology, and computational biology. The combination of high citation counts, sustained citation velocity, and above-average year-normalized citation impact indicates that African researchers are contributing not only quantitatively but also qualitatively to global P450 science. These findings therefore provide additional evidence supporting the conclusion that African-led P450 research has generated internationally recognized scientific contributions that continue to shape research across multiple disciplines.

### 3.7. Under-Researched Areas and Knowledge Gaps

Despite considerable progress in African P450 research, several important knowledge gaps remain. One major limitation identified in this study was the relatively small number of large-scale clinical pharmacogenomics investigations. Although some studies examined P450 polymorphisms, most were limited to targeted gene analyses and small sample sizes. Given the exceptionally high genetic diversity within African populations, expanded pharmacogenomic research is essential to improving precision medicine, drug safety, and individualized therapeutic strategies across the continent.

Another major gap was the limited use of advanced omics and systems biology approaches. Only a few studies incorporated transcriptomics, proteomics, metabolomics, or integrated multiomics analyses. Similarly, structural biology and computational enzymology studies involving molecular docking, molecular dynamics simulations, cryo-electron microscopy, or rational enzyme engineering were rare. These findings suggest that African P450 research still relies largely on conventional biochemical and molecular approaches, likely due to limited access to advanced research infrastructure and computational resources.

Industrial biotechnology, environmental toxicology, and plant P450 research were also poorly represented. Few studies focused on synthetic biology, industrial biocatalysis, environmental biomonitoring, aquatic toxicology, or plant CYPs involved in secondary metabolism and crop improvement. Given Africa’s rich microbial and plant biodiversity, together with increasing environmental and agricultural challenges, these areas represent important opportunities for future research expansion and innovation.

## 4. Conclusions

African P450 research has expanded substantially since the first African-led publication in 1987, with strong growth observed after 2011. South African researchers emerged as the dominant contributors, although meaningful participation from researchers across multiple African countries demonstrates continent-wide engagement in P450 science. African researchers established important international collaborations and contributed significantly to global knowledge in pharmacogenomics, steroidogenesis, vector biology, microbial biotechnology, biosensors, and natural-product metabolism. The study further revealed that African P450 research is strongly focused on biomedical and toxicological applications, particularly drug metabolism and infectious disease-related challenges relevant to the continent. Beyond publication productivity, the citation analysis demonstrates that African-led cytochrome P450 research has achieved substantial international scientific visibility. Collectively, the 558 publications accumulated more than 20,500 Google Scholar citations, with nearly one-third exceeding the average citation impact of papers published in the same year. These findings indicate that African researchers are not only contributing an increasing number of publications but are also producing research that is widely recognized and utilized by the international scientific community. Despite these advances, major opportunities remain in clinical pharmacogenomics, systems biology, structural biology, industrial biotechnology, and integrated omics research. Strengthening research infrastructure, computational resources, funding opportunities, and both intra-African and international collaborations will be essential for expanding the impact of African P450 research. Overall, this work highlights Africa’s important and growing role in advancing cytochrome P450 science and provides a framework for future research development across the continent. Future studies could extend the present work by conducting comparative bibliometric analyses of cytochrome P450 research across Asia, South America, Europe, and Australia to situate African scientific contributions within a broader global context.

## Figures and Tables

**Table 1:** Notable contributions by African researchers to P450 research. The examples presented are illustrative and not intended to represent a ranking or exhaustive list of contributors.

Researcher Name	Affiliation	Research Contributions
Khajamohiddin Syed	University of Zululand, South Africa	Contributed to African and global P450 research through genome-wide annotation, comparative genomics, structural biology, and evolutionary analyses of cytochrome P450 enzymes across fungi, bacteria, archaea, oomycetes, cyanobacteria, and microalgae [23,24,25,26,27]. His studies advanced the understanding of microbial P450 diversity, conserved motifs, structure–function relationships, secondary metabolism, and important CYP families, including CYP53, CYP51, CYP102, CYP107, CYP109, CYP121, CYP125, CYP128, CYP139, and CYP5619 [28,29,30,31,32,33,34,35,36,37,38,39,40]. He also contributed bioinformatics resources, including P450Atlas and comparative analyses of P450–ferredoxin interactions, strengthening African contributions to microbial P450 genomics, structural biology, and computational biology [1,41].
Amanda C Swart	University of Stellenbosch, Cape Town, South Africa	Contributed to steroidogenesis research through studies on the structure–function relationships of CYP11A1 and CYP17A1, improving understanding of steroidogenic P450 enzymes [42,43,44,45,46,47]. Her research also examined the effects of indigenous South African medicinal plants on steroidogenic P450 enzymes and adrenal hormone production, and advanced knowledge of C11-oxy steroids and backdoor androgen pathways [48,49,50,51,52,53,54,55,56,57,58,59,60]. Collectively, these studies established important African contributions linking mechanistic enzymology with endocrinology, natural products, and steroid metabolism [61,62,63].
Pieter Swart	University of Stellenbosch, Cape Town, South Africa	Contributed to adrenal P450 enzymology, steroid metabolism, and cytochrome b5 function through studies of CYP17 and steroidogenesis in South African livestock [64,65,66,67,68,69,70,71]. His work identified CYP17 variants associated with altered steroid production and hypocortisolism and clarified the regulatory roles of cytochrome b5 [72,73,74,75,76]. These studies strengthened African contributions to molecular endocrinology and steroid biochemistry [77].
Charles S Wondji	Centre of Research in Infectious Diseases, Cameroon & Liverpool School of Tropical Medicine, UK	Contributed to understanding insecticide resistance in African malaria vectors by identifying P450 genes responsible for pyrethroid metabolism and resistance, including *CYP6P9a*, *CYP6P9b*, *CYP6M2*, *CYP6P4*, *CYP6Z1*, and *CYP6P3* [78,79,80,81,82,83]. His work also led to molecular diagnostic tools for monitoring resistance and improving vector-control strategies across Africa.
Emmanuel Iheanyichukwu Iwuoha	University of the Western Cape, South Africa	Advanced African P450 biosensor research by integrating electrochemistry, nanotechnology, and biocatalysis to develop sensitive P450-based biosensors for pharmaceutical, environmental, and biomedical applications [84,85,86,87,88,89,90]. His work demonstrated the value of P450 bioelectrodes in drug screening, therapeutic monitoring, diagnostics, and analytical biotechnology.
Martie S Smith and Dirk J Opperman	University of the Free State, South Africa	Contributed to microbial P450 biotechnology through studies on fungal and bacterial P450 monooxygenases for industrial biocatalysis [91,92,93,94,95,96,97,98]. Their work advanced understanding of CYP153, CYP505, CYP53, and related enzymes, emphasizing enzyme engineering, regioselective hydroxylation, whole-cell biocatalysis, and sustainable oxyfunctionalization. These studies established Southern Africa as an important contributor to industrial P450 biotechnology.
Collet Dandara	University of Cape Town, South Africa	Contributed to African P450 pharmacogenomics by investigating genetic variation in P450 enzymes and its impact on drug metabolism, particularly in HIV pharmacotherapy [99,100,101,102]. His research also examined herb–drug interactions involving African medicinal plants and the molecular regulation of drug-metabolizing enzymes [103,104,105,106]. Collectively, these studies strengthened African contributions to personalized medicine and public health pharmacology.
Collen Masimirembwa	University of the Witwatersrand, South Africa & African Institute of Biomedical Science and Technology, Zimbabwe	Contributed to African pharmacogenomics by demonstrating how CYP2B6 and CYP2D6 genetic variation influences drug metabolism and treatment outcomes in African populations [107,108,109]. His work also examined the impact of P450 polymorphisms on tamoxifen metabolism in breast cancer patients, advancing precision medicine and personalized drug therapy [110,111].
Michael Sean Pepper	University of Pretoria, South Africa	Contributed to African pharmacogenomics by investigating P450 genetic diversity and genotype–phenotype relationships relevant to drug therapy in African populations [112,113,114,115,116]. His work strengthened the translation of pharmacogenetic knowledge into precision medicine and optimized drug therapy for African populations.
Lizette Leonie Koekemoer	National Institute for Communicable Diseases & University of Witwatersrand, South Africa	Contributed to African P450 research by investigating P450-mediated insecticide resistance in *Anopheles* mosquitoes, particularly *A. funestus* and *A. arabiensis* [117,118,119,120,121]. Her studies identified resistance-associated P450 genes and linked gene expression to insecticide resistance, providing important insights for malaria vector surveillance and control.
Scott Hazelhurst	University of the Witwatersrand, South Africa	Contributed to African P450 pharmacogenomics through the development of computational approaches for accurate genotyping of P450 genes in African populations [122,123,124,125].

Note: The researchers included in Table 1 were selected to provide representative examples of major scientific contributions across the principal areas of African P450 research. In addition to the ten researchers shown in Figure 5, Masimirembwa C and Hazelhurst S were included because of their significant contributions to African P450 pharmacogenomics, bioinformatics, and the application of P450 research to human health and precision medicine.

**Table 2:** Thematic classification and distribution of African P450 research publications.

Research Category	Approximate Number of Articles	Approximate Proportion	Major Research Focus
Drug metabolism, pharmacology, and toxicology	~210–220	~38–40%	Xenobiotic metabolism, drug interactions, hepatotoxicity, pharmacokinetics, inhibitor studies, antimalarial and anticancer metabolism.
Molecular biology, genomics, and P450 expression studies	~135–145	~24–26%	P450 gene cloning, polymorphisms, transcriptomics, recombinant expression, evolutionary analysis, and regulation.
Biochemistry, enzymology, and structural biology	~90–100	~16–18%	Enzyme kinetics, catalytic mechanisms, heme chemistry, substrate specificity, and structural characterization.
Clinical, medical, and disease-associated studies	~40–50	~7–9%	Cancer susceptibility, steroid metabolism, infectious diseases, and ADME-related clinical studies.
Vector biology and insecticide resistance	~25–35	~5–6%	Mosquito resistance mechanisms, detoxification pathways, and malaria vector control.
Microbial/fungal P450s and biotechnology	~20–30	~4–5%	Biocatalysis, fungal P450s, biodegradation, microbial metabolism.
Environmental toxicology and ecotoxicology	~10–15	~2–3%	Aquatic biomarkers, pollution monitoring, pesticide exposure studies.
Plant P450s and natural-product metabolism	~10–15	~2–3%	Plant secondary metabolism, phytochemical biotransformation, and medicinal plant studies.
Biosensors, analytical methods, and applied technologies	~5–10	~1–2%	Biosensor development, analytical assays, diagnostic approaches.

## Data Availability

All data supporting the results of this study are provided in the Supplementary Materials (Tables S1–S6).

## Supplementary Materials

The following supporting information can be downloaded at https://www.mdpi.com/article/10.3390/biology15151242/s1, Table S1: Analysis of African-led P450 research from PubMed from 1987 to 2025; Table S2: Analysis of African P450 researchers’ co-authored articles from PubMed from 1987 to 2025; Table S3: Analysis of non-African P450 researchers’ association with African institutions; Table S4: Article with no P450 aspects; Table S5: Information on non-African researchers who led P450 research articles on African problems; Table S6: Citation impact analysis of African-led cytochrome P450 publications. The table summarizes Google Scholar citation counts, citation velocity, and year-normalized citation impact (YNCI) for each publication included in the bibliometric analysis.
